# Data Processing in Functional Near-Infrared Spectroscopy (fNIRS) Motor Control Research

**DOI:** 10.3390/brainsci11050606

**Published:** 2021-05-09

**Authors:** Patrick W. Dans, Stevie D. Foglia, Aimee J. Nelson

**Affiliations:** 1 Department of Kinesiology, McMaster University, Hamilton, ON L8S 4K1, Canada; danspw@mcmaster.ca; 2 School of Biomedical Engineering, McMaster University, Hamilton, ON L8S 4K1, Canada; foglias@mcmaster.ca

**Keywords:** fNIRS, data processing, functional near-infrared spectroscopy, processing techniques, motor control

## Abstract

FNIRS pre-processing and processing methodologies are very important—how a researcher chooses to process their data can change the outcome of an experiment. The purpose of this review is to provide a guide on fNIRS pre-processing and processing techniques pertinent to the field of human motor control research. One hundred and twenty-three articles were selected from the motor control field and were examined on the basis of their fNIRS pre-processing and processing methodologies. Information was gathered about the most frequently used techniques in the field, which included frequency cutoff filters, wavelet filters, smoothing filters, and the general linear model (GLM). We discuss the methodologies of and considerations for these frequently used techniques, as well as those for some alternative techniques. Additionally, general considerations for processing are discussed.

## 1. Introduction

**Figure 1:**
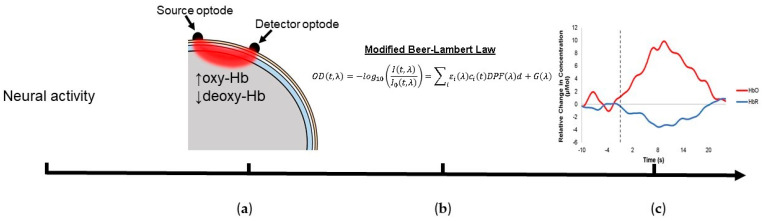
A graphical illustration of the stages of fNIRS data acquisition. (**a**) NIR light is projected along a banana-shaped path from the light optode through the scalp, skull, cerebrospinal fluid, and into the cortex. The light is absorbed, scattered, and reflected out of the head to the detector. (**b**) Changes in light intensity are related to concentration changes in hemoglobin through the modified Beer-Lambert law. This produces the hemodynamic response function (HRF). (**c**) Oxy-Hb is represented by the red line, whereas deoxy-Hb is represented by the blue line. The *x*-axis is time in seconds, and the *y*-axis is the relative change in concentration in micromolars.

Functional near-infrared spectroscopy (fNIRS) is a form of neuroimaging that utilizes light in the near-infrared range (700–1000 nm) to measure concentration changes in hemoglobin present in the cortex [1]. Neural activation is dependent on glucose and oxygen present in the cortical region. Following the onset of brain activity, a decrease in oxygenated (oxy-Hb) hemoglobin and an increase in deoxygenated (deoxy-Hb) hemoglobin occurs in the area of activity. Once the available substrate in the region is utilized, sustained brain activity is dependent on the ability of vascular channels to supply cortical regions with blood rich in oxygen and glucose [2]. Once these available substrates are reduced cerebral blood flow to that region increases through local arterial vasodilation, a process known as neurovascular coupling [3]. In fNIRS, photons of light are projected into the scalp by the source optode and pass through the skull and into the upper cortical regions. These photons are scattered and reflected as they travel through the head. Some of these photons are absorbed by the chromophores of oxy-Hb and deoxy-Hb [4]. The photons that are not absorbed are reflected and follow an elliptical path back to the surface of the scalp. These photons are measured using the detector optode [4]. As the concentration of oxy-Hb increases during neurovascular coupling, the intensity of the reflected light decreases. This process is a result of an increase in light photon absorption from the increased concentration of oxy-Hb in the activated region. The modified Beer–Lambert law is used to quantify changes in oxy-Hb and deoxy-Hb as a result of neurovascular coupling (see Figure 1b). In this equation, optical density (OD) is equal to the negative log of the attenuated light intensity (I) over the initial light intensity (I_0_). (t) represents time and (λ) represents the wavelength of light being used. This inverse relationship is equal to the sum of the molar extinction coefficient (ε_i_) multiplied by the concentration of hemoglobin (c_i_). These terms are multiplied by the differential pathlength factor (DPF), which accounts for the increase in distance that light travels due to light scatter, and the source-detector distance (d), where (i) represents all of the investigated chromophores. (G) represents the intensity of light lost due to scattering. Additionally, this equation accounts for the scattering of the light photons [5,6,7]. Concentration changes are determined by taking the inverse log of the light that was projected into the scalp by the light that was detected. Water is assumed to be a constant in this model as it will not change during neurovascular coupling (Figure 1) [5,8,9].

Unprocessed fNIRS data contain noise from different sources including physiological, instrumental, and motion that may conceal the task-related functional cortical signal. As such, revealing a functional signal requires pre-processing and processing of the collected fNIRS data. Treatment of data with pre-processing techniques allows for the removal of noise sources. Following noise removal, the hemodynamic response function (HRF) is derived from the optical signal using processing techniques. The implementation of sensible pre-processing and processing methods is of critical importance for the accurate detection of task-related cortical hemodynamic events.

The HRF used in fNIRS has shown to be comparable to the blood oxygen level dependent (BOLD) response from functional magnetic resonance imaging (fMRI) [10,11,12]. Due to its relatively low cost and portability compared to fMRI, fNIRS has gained significant attention and use over the last decade. The motor cortex has been the focus of many fNIRS studies due to its superficial location and proximity to the scalp [10,13,14,15,16,17]. Further, the portability of fNIRS makes it particularly useful in motor control research as it can be used to study complex motor paradigms where human movement is needed [18].

Due to the rising popularity of fNIRS, many different pre-processing and processing techniques exist leading to an increase in variety of fNIRS processing methodologies [18]. As a result, determining the most suitable pre-processing and processing techniques may be challenging. The goal of this review is to identify the most common fNIRS pre-processing and processing methodologies used in motor control research. Subsequently, the techniques identified as the most frequently used will be discussed in terms of their uses, considerations for utilization, and methodologies. Further, we discuss alternative, less common approaches to fNIRS processing that may benefit the field of motor control research. The information provided in this review is intended to assist researchers in determining the most appropriate techniques for a specific dataset. The results of our search criteria indicated that continuous wave fNIRS systems comprised the vast majority of the research that met the inclusion criteria. Therefore, the present review is largely focused on continuous wave fNIRS systems.

## 2. Materials and Methods

### 2.1. Literature Search Criteria and Data Extraction

The following PubMed headings were used in a PubMed literature search: ((((((((NIR) OR NIRS) OR fNIRS) OR fNIR) OR functional near-infrared spectroscopy) OR functional near-infrared spectroscopic) OR optical imaging system) OR optical topography) AND ((((((((motor) OR motor control) OR motor behaviour) OR motor behavior) OR motor function) OR motor coordination) OR motor activity) OR motor ability) AND (((((((((((((((((upper limb) OR lower limb) OR gait) OR locomotion) OR balance) OR ambulation) OR cycling) OR walking) OR standing) OR obstacle) OR dorsiflexion) OR plantarflexion) OR finger opposition) OR finger tapping) OR squeezing) OR grasping) OR manipulation) AND (“2010/01/01”[PDAT]: “2020/12/31”[PDAT]). Additionally, the NCBI filter function was used to narrow the search on the basis of the date of the study (1 January 2010 to 31 December 2020). Studies were also limited to those written in English and performed on humans. The following information was independently collected from each study by two graduate trainees (P.D., S.F.): first author, year of publication, experimental task, participant demographics, sample size, and processing approach.

### 2.2. Inclusion/Exclusion Criteria

**Figure 2:**
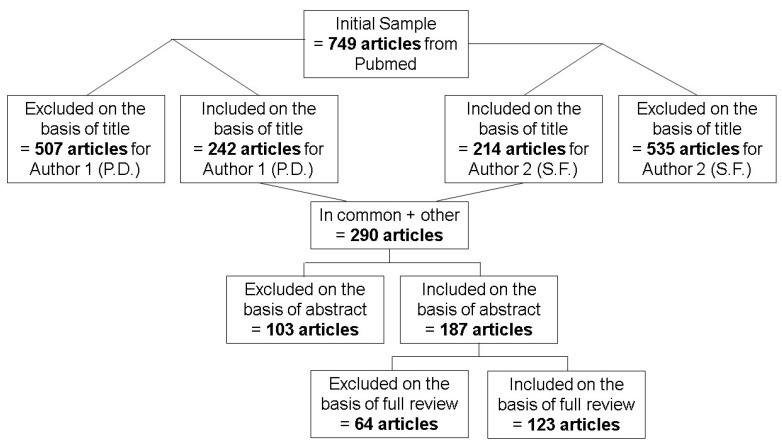
Figure depicting the study selection process for the review.

To have been included in this review, studies had to meet the following criteria: (a) be peer-reviewed articles; (b) use an ON/OFF task paradigm which alternates between motor task and rest periods; (c) use fNIRS in relation to, in combination with, or separate from other neuroimaging techniques on the cortex of the brain; (d) be performed in healthy human populations; and (e) report processing techniques for fNIRS data. These criteria were used to determine the inclusions at each stage of the review process (Figure 2).

## 3. Results

**Figure 3:**
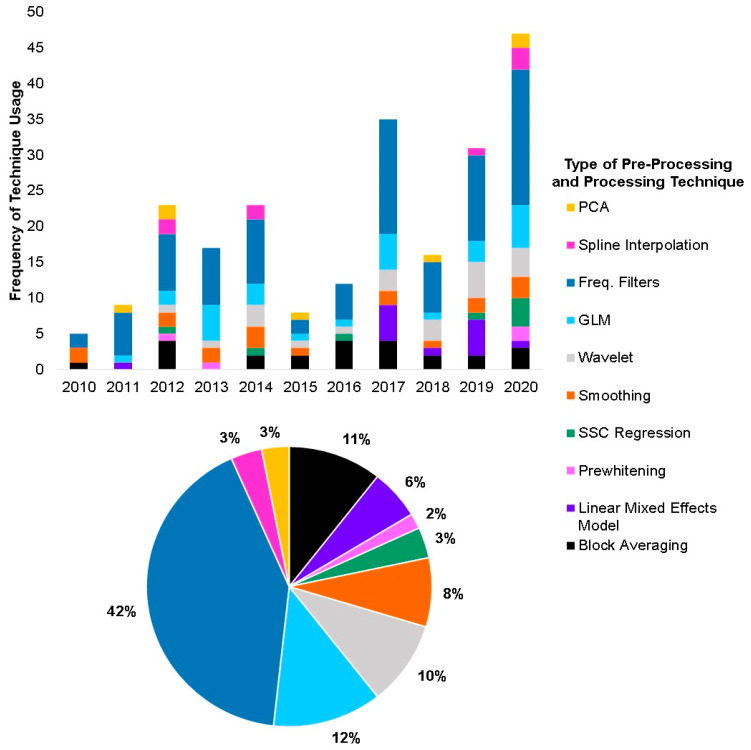
Technique usage over the past decade (2010–2020). (**top**) Each color in the stacked bars represents a type of pre-processing or processing technique. To see the studies utilizing each technique, please refer to Table S2 (Supplementary Materials). (**bottom**) Percent usage of pre-processing and processing techniques collapsed over the past decade.

Article information from the final sample of 123 articles is included in Table S1. Figure 3 displays the frequency of usage of different pre-processing and processing techniques between the years of 2010 and 2020. The papers that used specific pre-processing and processing techniques are included in Table S2. Studies are categorized on the basis of pre-processing and processing techniques employed, and as such, many studies appear more than once in the table, as more than one technique was typically utilized when pre-processing fNIRS data. Techniques were defined as frequently used if they were employed in ≥10 of the papers in the sample. The most frequently used pre-processing techniques were identified as bandpass filter, low-pass filter, high-pass filter, smoothing algorithms (moving average, Gaussian, Savitzky–Golay), and wavelet filtering. The most frequently used processing techniques were block averaging, linear mixed model, and the general linear model (GLM).

### 3.1. Stages of Data Pre-Processing and Processing

The primary goal of the pre-processing and processing of fNIRS data is to isolate the hemodynamic changes occurring in the vascular network of the gray matter. This is achieved by filtering raw data and estimating a HRF through modeling. These are referred to as pre-processing and processing, respectively. In pre-processing, the objective is to remove extraneous noise from the raw data. Noise can be classified as either systematic such as respiration, cardiac pulsation (heart rate), and changes in blood pressure [19,20,21] or motion artefact (MA) noise [9,22,23]. Noise removal techniques are applied prior to the HRF estimation. Frequently used pre-processing techniques include frequency filters, wavelet, and smoothing filters. Additionally, alternative methods such as pre-whitening can be used. Once the raw data has undergone pre-processing, methods are used to convert changes in light intensity to concentration changes in hemoglobin. Processing is used to compare baseline and task-related hemodynamic changes [24]. These can be separated into either general linear model (GLM) or non-GLM processing methods such as block averaging and linear mixed models.

### 3.2. Pre-Processing Techniques

#### 3.2.1. Systematic Noise Removal

Systematic noise can be introduced into the fNIRS signal from environmental/instrumental sources, cardiac pulsation, respiration, and cyclic changes in arterial blood pressure known as Mayer waves. Filtering can be employed for this noise from the fNIRS signal to be removed. Variation can be introduced into the fNIRS signal as a result of the type of pre-processing technique used [25,26,27]. Researchers must choose appropriate filters that remove systematic noise while preserving the functional hemodynamic signal. This section examines the most frequently used pre-processing techniques as well as alternatives that can be used to remove systematic noise.

#### 3.2.2. Low-Pass, High-Pass, and Bandpass Filters

There are two general types of frequency filters: infinite impulse response (IIR) and finite impulse response (FIR) filters. The mathematical equations for these two types of filters differ in their filter coefficients, which are calculated as the ratio between the sampling frequency of the system and the cutoff frequency of the filter [28].

The FIR filter type has filter coefficients that are composed entirely of inputs, whereas the IIR filter has filter coefficients that are comprised of both inputs and previous outputs of the filter. The output of the IIR filter can be *recursive* as it depends on both inputs and previous outputs [28]. FIR filters possess a linear phase, which allows for no phase distortion of the signal. IIR filters, however, have phase distortion, in that different frequencies have different levels of phase shift. This distortion can be avoided by using a zero-phase filter [29]. Pinti et al. [29] suggest using a high-order (>1000) FIR filter in place of an IIR filter due to the problem of phase distortion. Additionally, FIR filters are considered inherently stable as they always have a finite output for a finite input. IIR filters may not be stable, as the output could be finite or infinite [29,30].

**Figure 4:**
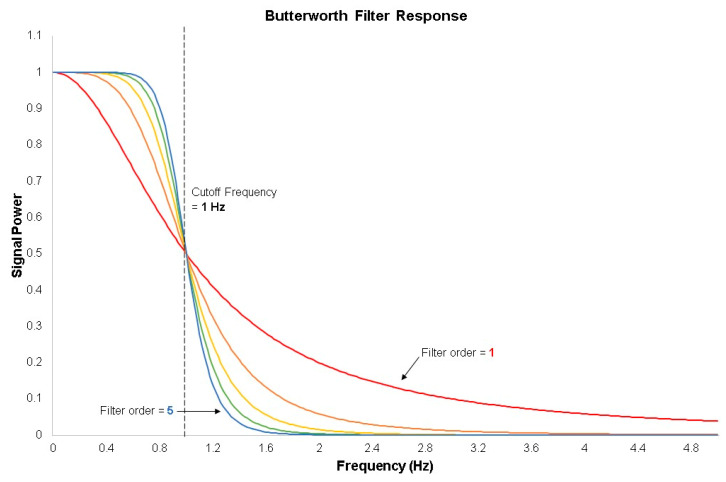
The magnitude-response of a low-pass Butterworth-type filter with a cutoff frequency of 1 Hz. Different colors represent different orders of filter. Red has a filter order of 1, orange has a filter order of 2, yellow has a filter order of 3, green has a filter order of 4, and blue has a filter order of 5. Higher order filters have steeper curves, and thus attenuate signals to greater degrees than lower order filters, except at the cutoff frequency. As signals increase in frequency, they are attenuated more, the degree to which is decided by filter design.

Filter order is another important characteristic. The higher the order of a filter, the greater the slope of the filter at the cutoff frequency (see Figure 4) [28]. In the filter’s equation, the number of filter coefficients represents the filter order, which increases as more coefficients are added. Consequently, filters with greater filter orders require more time to compute [28]. FIR filters need to be implemented with greater orders than IIR filters to obtain similar results [29]. Consequently, FIR filters require a greater amount of time to compute the output of the filter due to the greater number of terms in the equation.

A low-pass filter passes signals with a frequency lower than a selected cutoff frequency and attenuates signals with frequencies higher than the cutoff frequency [28]. Similarly, a high-pass filter passes frequencies higher than a cutoff while attenuating lower ones. The band-pass filter passes frequencies within a certain band, while outside the band, frequencies are attenuated. In these filters, the passband describes the range of frequencies passed through the bandpass filter, whereas the stopband describes the range of frequencies that are attenuated.

**Figure 5:**
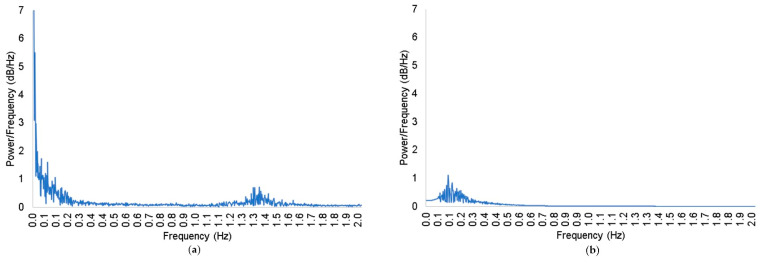
A comparison between FFTs of (**a**) unprocessed oxy-Hb concentration data and (**b**) bandpass filtered data (0.1–0.4 Hz, 3rd order IIR Butterworth filter). The *x*-axis represents frequency in Hz, and the *y*-axis represents power/frequency in dB/Hz. As can be seen, the higher frequencies in (**a**) (≈1.5 Hz, presumably related to HR) are significantly reduced in power when the bandpass filter is applied (**a**). What is less noticeable but still of note is the reduction in power of very low frequency oscillations (≈0.01 Hz) from nearly 110 dB/Hz to ≈0 dB/Hz (**b**).

These filters are used in fNIRS to attenuate high- and low-frequency physiological and instrumental noise. The low-pass filter is used to attenuate very high frequency noise arising from the environment such as extraneous light, and physiological noise such as cardiac pulsation and respiration. The high-pass filter is used to attenuate very low frequency oscillations, specifically those from baseline drift, which can arise from the gradual movement of the optodes on the scalp. The bandpass filter is a simple combination of a low-pass and high-pass filter, in that it passes a certain band of frequencies and attenuates the frequencies located outside of the band (Figure 5).

To implement these filtering techniques, one must choose the type of filter, filter order, and cutoff frequency (or frequencies). There are many different subtypes of these filters, the most common being the different IIR filters such as Butterworth, Chebyshev Types I and II, and the Elliptic filters, as well as the FIR filter [31]. Butterworth filters are designed to be maximally flat magnitude response filters, in that frequencies in both the passband and the stopband experience the least distortion possible [30]. Chebyshev Type I filters are designed so that frequencies beyond the cutoff frequency are much more sharply attenuated and monotonic (flat), while passband frequencies become slightly distorted [30]. Chebyshev Type II filters are designed such that the passband is as monotonic (flat) as possible, which then introduces distortion into the stopband [30]. Elliptic filters have equal distortions in both the passband and the stopband; however, these filters also have the highest rate of attenuation of the different filters for the same order [30].

When these filters are used, differences in physiology between populations and individuals may necessitate the adjustment of filter parameters. For example, athletes have lower resting cardiac pulsation than non-athletes [32]. In the case of a low-pass filter, the researcher would have to potentially lower the cutoff frequency to account for the lower resting heart rate of the athlete. For the higher resting heart rate of the non-athlete, the researcher could potentially use a higher cutoff frequency for the low-pass filter. Additionally, the type of task used can influence filter decisions. For example, heart rate and respiration rate increase during exercise vs. non-exercise motor tasks [33]. Therefore, implementing a filter with a “one-size-fits-all” cutoff frequency is not ideal. In consideration of these factors, applying a fast Fourier transform (FFT) to an fNIRS dataset will allow the researcher to visually inspect the data and determine the spectral location of noises within a dataset. Although no definitive parameters have been defined in the literature, Naseer and Hong [31] recommend a passband of 0.1~0.4 Hz to remove most physiological and instrumental noises from fNIRS data if the task period is 10 s in length.

These filters are quick and easy to implement, and are included in most fNIRS processing programs such as HomER2 [34]. As well, filtering techniques such as these can be useful since the frequencies related to physiology are usually known [31]. However, some researchers disagree with this notion and instead suggest that frequency characteristics can vary across time, location on the head, and participant [35]. Additionally, some types of frequency filters produce “ripples”, which affect the signal amplitudes of certain frequencies in the passband and/or the stopband [30]. As a result, some cortical data may be distorted, or some frequencies in the stopband may not be attenuated. Another related aspect of these filters is that frequencies in the stopband are not completely removed, but instead are only attenuated [31], still allowing some noise to pass through the filter. Even if noise does not penetrate the filter due to incomplete attenuation, some physiological noise (i.e., Mayer waves) can overlap in frequency with the cortical signal [35]. This overlap prevents the filter from completely removing noise while preserving signal. As such, other techniques have been created to better distinguish physiological noises from the cortical signal of interest, such as short-separation channel regression. Finally, with improper use of these filters, the cortical response may be affected. For example, if the cutoff frequency is set to remove noise in the range of the hemodynamic response (≈0.15 Hz for a 10 s task) [36], the user risks altering a portion of the response itself, either by attenuation or amplification of certain frequencies [35].

In summary, low-pass, bandpass, and high-pass filters are all pre-processing techniques that are employed to attenuate high and/or low frequency noise in fNIRS. Many different types and subtypes of these filters exist, which may distort noise or data depending on the type. Types of filter (IIR or FIR) can also differ in their time requirement for computation, which may impact decisions for usage in online scenarios. Individual physiological differences can also influence usage of these techniques, particularly regarding the cutoff frequency.

#### 3.2.3. Smoothing Filters

This technique is most frequently used to decrease the presence of high-frequency noise in fNIRS data. Smoothing filters can thus be a type of low-pass filter. However, the difference is the method by which smoothing filters reduce high-frequency noise. For example, the moving average filter smooths signals by averaging neighboring points and using that average as the new value of a point [37]. In the low-pass filter, however, lower frequencies are passed, and higher frequencies are specifically attenuated. There are many different types of smoothing algorithms, some of the most frequently used in our search being the moving average [38,39,40], Gaussian smoothing [41,42,43], and Savitzky–Golay smoothing filters [44,45].

Signals can be smoothed by either smoothing in the time domain or in the spatial domain. Time domain smoothing reduces the contribution of high-frequency noise in the data, whereas spatial smoothing averages signals from poor channels with the surrounding fNIRS channels, reducing the effect of the noisy channel while still preserving some of its signal [46]. The moving average type of smoothing works by averaging a number of data points together, reducing high-frequency fluctuations [47]. Gaussian smoothing involves a Gaussian weighting function, which multiplies the value of each point according to where it is on the distribution. The center of the Gaussian is set on one point, which is weighted along with the neighboring points. The distribution is then moved to the next point and the process is repeated [48,49]. Savitzky–Golay smoothing is mostly employed to smooth over spike MAs [50]. It can also be used to smooth physiological noise in the data; however, the reasons for why this filter is appropriate are unclear in this circumstance [51]. This type of filter uses a least-squares polynomial to fit the fNIRS data within a certain window while preserving some higher frequencies [52]. For more detail on the specific mathematics of this filter, see [50].

To smooth fNIRS data, the type of smoothing filter needs to be chosen. This choice will depend on the specific requirements for the filter. The moving average filter replaces values on the basis of the average of neighboring data points [37]. To use this filter, one needs to decide on a window around the point they wish to average. Many fNIRS studies appear to use a five second window to smooth data (see Table S2). It is unclear why these studies use five seconds specifically, as no reasons were given for the chosen parameter. Once the window is chosen, applying the filter requires computational processing for each data point. Gaussian smoothing is implemented much like the moving average filter—only the neighboring points around the point of interest are weighted according to a Gaussian distribution, instead of merely averaged [48,49]. The Savitzky–Golay filter uses a polynomial fitting function to approximate the values of the fNIRS waveform within a specific time window [50]. The fitting is performed with a least-squares fitting function of *2n + 1*, where n is the number of neighboring samples in the window and can be equal or greater than the order of the polynomial [53]. Jahani et al. [53] suggest choosing an *n* less than the number of samples (time points) of the hemodynamic response, otherwise the response may be smoothed itself.

There is not much information regarding considerations for smoothing techniques; however, these techniques are similar to low-pass filtering in that they “smooth over” high-frequency spikes in the data. In contrast to low-pass filters, smoothing techniques do not account for the frequency components of the noise—they operate in the time or spatial domains. In this sense, smoothing techniques do not account for frequency-related aspects of the signal and noise. However, this may be seen as an advantage of smoothing algorithms, as they do not assume certain frequencies only represent noise. As learned from the implementation of smoothing filters in electroencephalography (EEG), smoothing a signal too strongly can have adverse effects [37]. In the case of fNIRS, the hemodynamic response may become distorted and less obvious in the time course of the experiment.

### 3.3. Additional Techniques to Remove Systematic Noise, Pre-Whitening

There are multiple augmentations to the GLM, which can be employed to reduce noise in the data. One of these changes is known as pre-whitening. The premise of this technique relies on the fact that fNIRS data contains systematic physiological noise, which varies in a predictable way. Since this noise is predictable, there are certain frequencies that are over-represented in the noise distribution, which consequently violate one of the GLM assumptions for a normal distribution of noise [54]. As such, pre-whitening can be implemented to estimate this noise and subsequently reduce the weights of the over-represented frequencies in the noise distribution. Specifically, an autoregressive model is fit to the residual noise from a first pass of the GLM. This model is then applied to both sides of the GLM equation to “whiten” the data (Equation (1)). A different number of passes can be completed with repeated stages of estimation and whitening, such as in Barker et al. [55], where this autoregressive process is repeated until convergence.

(1) W·Y=W·X·β+ε

One caveat to using this method is that it estimates the noise, and as such noise can be over- or under-estimated, potentially leading to bias [56]. However, the version of this technique created and implemented by Barker et al. [55] has been shown to be quite effective in controlling Type 1 error, and has improved sensitivity-specificity in comparison to ordinary least squares when combined with an iteratively-reweighted least squares solving method.

### 3.4. Motion Artefact Correction, Wavelet Filter

MAs can be introduced into the fNIRS signal by head movements that cause the source detector pair to shift relative to the scalp. Visually, MAs can present as rapid and very large changes in magnitude (spikes) relative to the baseline data. These spikes can be several orders of magnitude larger than the tissue-related hemodynamic changes [57]. Additionally, due to the movement of the source detector pairs on the scalp, the baseline fNIRS signal can shift. This can cause an artificially inflated positive correlation between oxy-Hb and deoxy-Hb [22,58]. These large spikes can be identified through qualitative visual inspection. Once identified, the segment of data containing the MA can be removed from the overall signal [59]. Mathematically based filtering methods can also be used to remove MA from the data. No standard MA removal technique has been identified in the literature. As such, variability in the fNIRS signal can arise depending upon the MA correction tool chosen for the experiment. This section will discuss the most commonly used techniques as well as suggest alternatives for MA correction.

**Figure 6:**
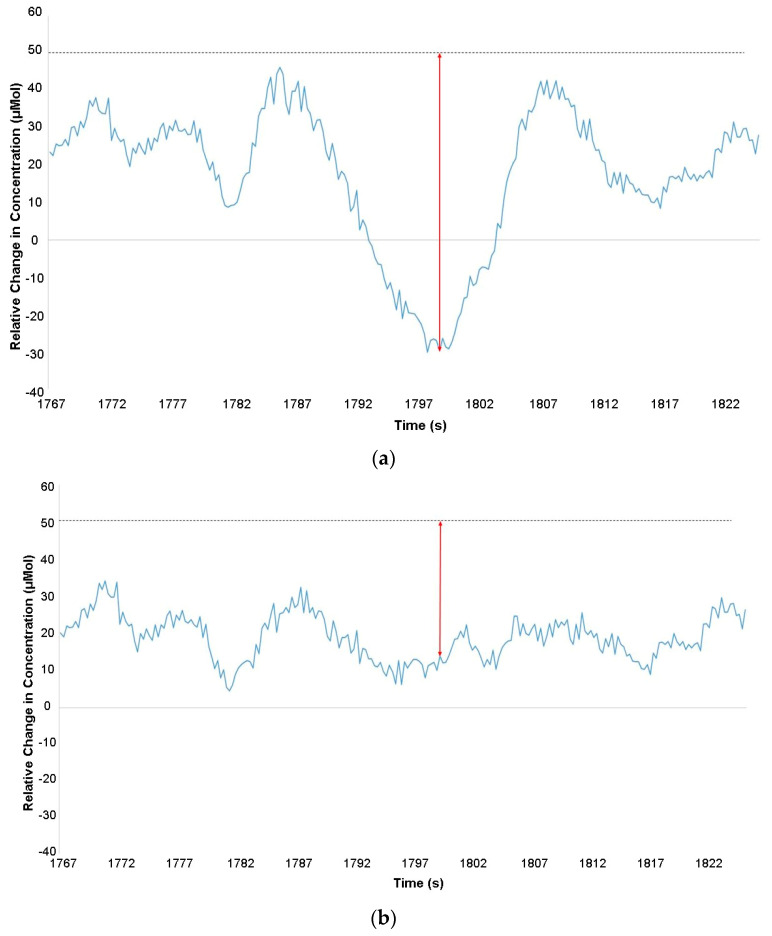
A comparison between unprocessed oxy-Hb concentration data (**a**) and data filtered with a discrete wavelet transform (IQR = 1.5) [60]. The *x*-axis represents time in seconds, and the *y*-axis represents relative change in oxy-Hb concentration in micromolar. A negative spike at ≈1800 s is first at an amplitude of ≈ −30 μMol in graph (**a**), however once filtered, it was reduced to a magnitude of ≈14 μMol in graph (**b**).

Wavelet filters are used to filter out different types of noise, but mostly spike MAs (Figure 6) [60]. Wavelet filtering is based on the premise that cortical signal is composed of different frequencies than MAs [60]. Wavelet filtering begins with the base mother wavelet, which is scaled and translated to create daughter wavelets [61]. The fNIRS recording is then decomposed using these daughter wavelets. Wavelet coefficients (expressions) describe how well the wavelet transform represents the fNIRS recording. The greater the number of wavelet coefficients, the better the wavelet transform can represent the full signal. These wavelet coefficients are organized into a distribution, associated with the scaling and translation parameters [61]. MAs are outliers in this distribution because of their differences when compared to cortical signal, and can therefore be removed [60].

There are different types of wavelet transforms, including the discrete wavelet transform (DWT) [58], the continuous wavelet transform (CWT) [62], and the minimum description length wavelet (wavelet-MDL), which is a specific DWT for reducing global physiological trends [63]. All wavelet transforms are derived from a mother wavelet, which is scaled and translated to produce the daughter wavelets [30]. The difference between the CWT and the DWT lies in the manner that the daughter wavelets are derived from the mother wavelet.

Daughter wavelets in the DWT are derived from specific methods, in other words by discretizing the scale, translation, time, and setting parameters of the mother wavelet [30]. For example, powers of 2 could be used to scale and translate a mother wavelet [58]. In DWTs, the number of wavelet coefficients required for full representation of the original signal is the same as the number of time points in the dataset. The discrete wavelet-MDL detrending algorithm can be used to remove spike MAs, as well as global trends in the data related to physiological activity [63]. It does this by estimating the number of wavelet coefficients required to fit the wavelet transform to the data, and then using the minimum number. In other words, if there are many different but viable ways to describe the data, use the simplest way [63].

In the CWT, there is less of a restriction on scaling and time shifting factors for the daughter wavelets than in the DWT [30]. The daughter wavelets can consist of any combination of scaled differences and translations of the mother wavelet. This means that redundancies may arise from CWTs due to oversampling; however, the flexibility of the scaling and translation parameters can make small changes in the data easier to interpret if used to a greater extent [61]. Consequently, the number of wavelet coefficients that may be used to describe the full signal in the CWT is much greater than the number of time points in the signal.

Generally, the utilization of the wavelet technique requires the researcher to not only choose which type of wavelet transform to apply (discrete, continuous), but also which mother wavelet to use and the scaling and translation parameters for the daughter wavelets. There are many different mother wavelets. For example, the wavelet transform described in Molavi and Dumont [60] designed specifically for fNIRS data uses the Daubechies mother wavelet [64]. Once the scaling and translation parameters are chosen for all wavelets, the wavelets are compared to the fNIRS data, and the data are decomposed into wavelet coefficients using the daughter wavelets. The decomposition allows for different frequencies in the original signal to be seen at different times, at which point frequency components relating to motion can be removed [58]. The wavelet coefficients, formed from the different daughter wavelets and their interactions with the data, are assumed to form a gaussian probability distribution [23]. In this distribution, wavelet coefficients around the zero mean with low variability are assumed to represent the slow frequency hemodynamic response, while those around the edges describe the highly variable, high-frequency MAs. The probability threshold, α, is then set by the researcher to know which wavelet coefficients to remove from the distribution, i.e., if a coefficient does not meet the probability threshold, then it is labeled an artifact and is decreased in signal amplitude [23]. After the outliers are reduced in signal amplitude, the rest of the coefficients are combined to form the original waveform, without artifacts [60].

This method of filtering relies on the assumption that MAs oscillate much faster in time than fNIRS hemodynamic signals [60]. As such, MAs that result from slower movements over time are not identified by this filter. As well, the type of MAs present in the researcher’s fNIRS data may depend on the population. For example, young infants are known to move even in their sleep and cannot be instructed to keep still [62]. Additionally, infants are known to make spontaneous movements during long stimulation periods [65]. These spontaneous movements are likely to result in fast changes of the hemodynamic signal, in the form of baseline shifts or spike artefacts. Adults, on the other hand, may be less likely to produce MAs considering the simple fact that they can be instructed not to move outside the confines of the task presentation. Wavelet transforms are useful in that they can localize fast signal changes and can separate the signal into different frequencies at different times, which allows for the removal of solely motion-related components. However, they are not good for removing artefacts with slower oscillations [58]. With improper usage, MAs are not removed efficiently from the data. Specifically, if the threshold criterion is too strong or weak for MA removal, the researcher risks removing too much signal or too little artefact.

In summary, wavelet filters involve the decomposition of an fNIRS recording into its constituents. This technique is useful for removing MAs and physiological noise, depending on the type of wavelet filter used. The two types of wavelets are CWT and DWT, which decompose the fNIRS recording on the basis of non-discretized or discretized wavelet parameters, respectively.

### 3.5. Alternatives for Motion Artefact Correction: Principal Component Analysis

An alternative approach that can be used to reduce MAs is principal component analysis (PCA). PCAs operate under the assumption that MAs present in the data would occur in all channels and produce similar temporal variation. The PCA technique arranges the data in a matrix containing the number of time points by the number of channels. A set of orthogonal vectors is then derived from this matrix in decreasing order on the basis of the amount of variance present in each vector. The MA produces the greatest amount of variance present across all channels, which is captured by the principal components. A percentage of variance present in all channels is then used to remove a number of components from the data [66]. Once these components have been removed, the remaining components are used to reconstruct the signal. Although the MA occurs at a specific time, the PCA analysis is not specific to this time point and is rather applied to the entire duration of the dataset. As such, functional signal that is not impacted by MAs can be removed [66]. One way of preventing the overcorrection of the data is by using a targeted principal component analysis (tPCA) [66]. A tPCA operates on the same principles as described above but is only applied to the section of data that contains the MA. The tPCA has been shown to be more robust in preserving hemodynamic response contaminated by MA compared to wavelet-based filtering and spline interpolation [26].

### 3.6. Processing Techniques

#### 3.6.1. General Linear Model

The GLM is a method of statistical modelling for fNIRS data. It has previously been used to model the fMRI BOLD response [67], and has been adopted due to the similarity between the BOLD and HRF. The GLM utilizes predictors to describe the largest sources of variability within the fNIRS data [67]. For example, the researcher would input their task timings into the model, along with the predictor that describes the hemodynamic response, either through estimation or assumption of the shape of the HRF. Some studies model the hemodynamic response with a linear combination of gamma functions as a predictor [68,69,70], assuming the shape of the hemodynamic response function. Other studies use a deconvolution procedure [71], which estimates the hemodynamic response with a series of Gaussian functions spaced in increments along the task period.

In its simplest form, the GLM is represented by a linear equation (Equation (2)), in which the amplitude of the hemodynamic response in one channel (*Y*) is equal to the predictor (*X*) multiplied by the “weight” of that predictor (*β*) plus the error term (*ε*).

(2) Y=X·β+ε

Predictors are given weight in the model regarding how much that predictor contributes to the variability of the signal. In other words, if the researcher’s estimate/assumption of the shape of the hemodynamic response is correct, then that estimate will be given a higher weight by the model. The error term in the equation represents all noise in the recording, consisting of physiological, instrumental, and motion noise.

The GLM has assumptions regarding the data and the noise in the system. These assumptions are as follows [67]:

1. Task responses are non-stochastic (non-random) and are the same across trials of the same task.
2. Noise is independently and identically distributed, with a mean of zero and with some amount of variance around that point.
  - Noise is homoscedastic, meaning there is noise from only one distribution in the data.
  - Noise is not serially correlated, meaning that past noise does not affect future noise.
3. Predictors are not linear derivations of each other.

To use this technique, a researcher needs to decide on the method of inference/estimation of the hemodynamic response. As mentioned above, many fNIRS studies assume the shape of the hemodynamic response with canonical gamma functions. Different convolutions of gamma functions are used to assume the shape of the canonical hemodynamic response [57]. While this method of the GLM is useful if the shape of the response is already known, assuming the shape could potentially lead to modelling errors as the response can change between recordings [72]. Another potential method is deconvolution [67], which instead estimates the shape of the hemodynamic response. After the method is chosen, the predictors are put into the GLM, which then estimates the weights of each of the predictors (i.e., how much they contribute to variability) and if they are significantly different from zero [67]. Some programs, such as HomER2, have additional parameters to control such as the option to include short-separation channels (SSC) as predictors or the option to change the GLM solving method [34].

An additional consideration for the GLM is that the researcher can avoid the uncertainty of the differential path length factor (DPF) [57], a term used to correct for the extra distance that NIR light travels in the cortex due to light scatter from biological tissues [73]. The DPF is a highly variable parameter because it can change between different ages and populations of participants [74], as well as between brain regions [75]. Group analysis of fNIRS data can also be easily completed using a multi-level GLM analysis [57]. However, fNIRS data seem to violate many of the GLM assumptions, particularly regarding the contents of the noise [67]. MAs and systematic physiological noise violate the assumption that noise is independent [55], thus leading to biased results from the GLM. Specifically, noise comes from multiple distributions and is not independently distributed [24].

There are many different ways to solve the GLM equation, which can affect the final results. Specifically, a least squares approximation is used to solve the model by correcting for differences between the model’s prediction and the actual fNIRS data [76]. Ordinary least squares (OLS) and iteratively reweighted least squares (IRLS) are just some of the different methods used to solve the GLM. OLS operates under the assumptions that the noise has a zero-centered mean, is independent, and is identically distributed. However, the assumption of a zero-centered mean can be violated if the data contains MAs, which produces heavy tailed noise [55]. As well, the assumption of independence is violated as noise in fNIRS data contains serially correlated errors [77]. In contrast, IRLS is a more robust solving method in which the GLM equation is first solved with weighted least squares (WLS), but then is iteratively solved after recalculating the βs until the point when the βs do not change a significant amount [55]. These are just two of the methods that could be used to solve the GLM equation; however, we chose to present information about these two methods as they were the only ones used in our sample.

To summarize, the GLM is a statistical technique used to model the cortical signal recorded with fNIRS. It is simple and effective, as it assumes the recording is simply the linear combination of multiple regressors, representing task-related cortical signal and task-unrelated noise. The GLM also has multiple assumptions, of which fNIRS violates many. However, with proper removal of noise-related artifacts, assumptions can be met and therefore the true hemodynamic response can be assessed.

#### 3.6.2. Block Averaging

Block averaging is a frequently used fNIRS processing method [29]. This processing method uses simple weighted averaging for fNIRS signals in blocks of task periods [78]. After this point, statistical procedures may be performed to assess if the HRF is different from baseline signals. This method is very simple and thus may be easier to implement for newer fNIRS researchers [79]. However, the GLM is preferred to this method as the HRF can be derived simultaneously along with the removal of noise components [80]. Additionally, the estimation of the response tends to be more accurate and robust utilizing the GLM when compared to block averaging [81,82]. Another consideration for block averaging is that it does not utilize the time course of the HRF, which is important in NIRS analysis [57]. The GLM, however, is more statistically powerful than block averaging as it considers the entire time-course of fNIRS data [29].

#### 3.6.3. Linear Mixed Models

A linear mixed effects model is another potential processing method for fNIRS data. This model, like the GLM, assumes that the fNIRS time-series is a linear combination of regressors [57]. However, the additional aspect of this method is that it also includes a term for random effects, meaning it accounts for both within- and between-subject variability [83]. One advantage to using this type of model is that parameters unique to single participants can be evaluated. Additionally, this method can be used to model temporal changes non-linearly [84]. One potential disadvantage of using this model is the restricted maximum likelihood (ReML) that is used to estimate the noise in the data [55].

## 4. Discussion

The goal of this review was to identify the most common fNIRS processing techniques in the motor control field and to describe them in terms of their uses, methodologies, and methodological considerations. These techniques were identified as bandpass filters, low-pass filters, high-pass filters, smoothing algorithms (moving average, Gaussian, Savitzky–Golay), wavelet filtering, block averaging, linear mixed models, and the GLM. From the dataset, it is apparent that a large variety of pre-processing and processing techniques exist in the motor control field.

**Figure 7:**
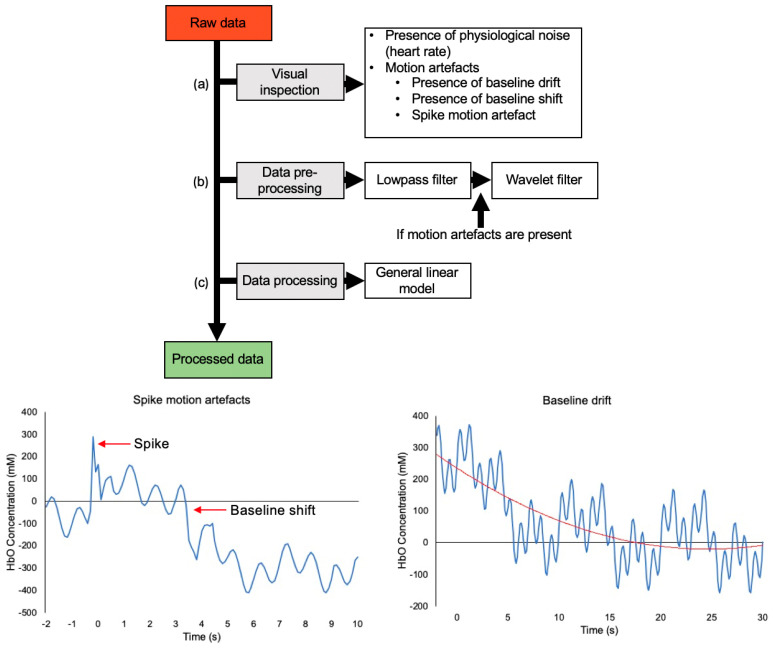
Data processing pipeline composed of the most common techniques over the past decade. (**a**) Raw data should first be inspected for the presence of physiological noise and MAs. The presence of physiological noise in the data shows that optodes are coupled to the scalp. Different types of MAs can arise in fNIRS data. Spike MAs and baseline shifts are caused by rapid changes in optode position across the scalp. Baseline drift can occur due to slow, constant movement of optodes across the scalp. (**b**) Data are subsequently subjected to pre-processing. The most common pre-processing technique identified was the frequency filter. Within frequency filters, low-pass filters were the most common. If the data possesses MAs, MA correction methods can be applied to the data in the pre-processing stage. The most common technique used for MA correction was found to be the wavelet filter. (**c**) Once the data have been pre-processed, further processing techniques can be applied to derive the HRF. The most common technique used for this purpose over the past decade was the GLM.

Figure 7 displays the common pre-processing and processing steps used in fNIRS studies in the past decade. As such, when choosing techniques for a processing stream, it is important to take the specific characteristics of the fNIRS dataset into account. For example, if the dataset does not include spike MAs, utilizing a wavelet filtering technique would be unnecessary as it specifically removes those artifacts [60]. Further, both physiological and motion characteristics can change between individuals, populations, tasks, and brain regions [15,35,74,75]. Therefore, the processing stream should be personalized for each dataset to account for differences in physiological, motion, instrumental noise, and variation in fNIRS responses to different tasks.

The improper use of a technique could result in the distortion of the fNIRS response (e.g., over-smoothing fNIRS data) [37] and possibly the incomplete removal of noise (e.g., low-pass filtering with a high cutoff frequency). The creation of a processing stream specific to the researcher’s dataset thus requires an in-depth understanding of the different processing techniques available, as well as their parameters and methodologies. Additionally, knowledge of the different techniques is important for the assessment of other studies in the field. For example, the researcher can assess the validity of other studies on the basis of their processing stream. This aspect is important for the progression of the field, as it ensures that high-quality research is being performed in both the researcher’s own work and others.

There are many different fNIRS processing techniques available to use, and the most common techniques may not necessarily be the best. For example, bandpass, low-pass, and high-pass filters could be replaced with SSC regression, which has been shown to be effective in reducing physiological noises [85,86]. SSC is used to mitigate the influence of scalp blood supply on the fNIRS signal. The scalp blood supply can affect signal by absorbing photons of light and causing changes in light intensity that are unrelated to hemodynamic changes in the cortex [87]. Using a source detector spacing of less than 30 mm allows for the exclusive detection of hemodynamic changes present in the scalp. These data can be projected onto the baseline signal to regress signal unrelated to functional changes in the cortex. SSC measurements are available with many devices, such as those from Artinis, Hitachi, and TechEn.

This review presents information that will help guide new fNIRS researchers regarding their processing; however, it is not without its limitations. This review is limited to papers in the decade of 2010–2020. As such, papers published outside of this range were not examined, and any information they may have provided regarding fNIRS pre-processing and processing was not considered. However, the focus of this review was not to examine all fNIRS pre-processing and processing, but instead to examine more recent methodologies to inform future studies. As the fNIRS field continues to grow, new techniques are constantly being developed. Focusing on studies in the last decade has allowed for the most recent and relevant techniques to be captured. Another limitation of this review is that studies outside of the motor control field were not included. Although the results of this review are derived from studies investigating human movement and motor control, similar processing can be applied to studies of human cognition or other fNIRS applications. These include frequency filters, motion artefact correction, HRF modelling, and the common processing shown in Figure 7. However, the content of this review is derived from the most common techniques used in motor control research. Future researchers may choose to explore other fields such as using fNIRS for cognitive research and expand their search criteria to encompass a more complete review of fNIRS pre-processing and processing methodologies.

## 5. Conclusions

FNIRS contains different types of noise in comparison to other neuroimaging modalities, requiring the implementation of specific techniques to remove such noise. As well, pre-processing and processing should account for differences in the noise due to time, ROI, and population. The information in this review contained in Table S1 [88,89,90,91,92,93,94,95,96,97,98,99,100,101,102,103,104,105,106,107,108,109,110,111,112,113,114,115,116,117,118,119,120,121,122,123,124,125,126,127,128,129,130,131,132,133,134,135,136,137,138,139,140,141,142,143,144,145,146,147,148,149,150,151,152,153,154,155,156,157,158,159,160,161,162,163,164,165,166,167,168,169,170,171,172,173,174,175,176,177,178,179,180,181,182,183,184,185,186,187,188,189,190,191,192,193,194,195] benefits the field by providing insight on frequently used techniques, and alternatives for those techniques to new fNIRS researchers. This information may aid both current and future fNIRS researchers to provide a basis for their own pre-processing and processing.

## Supplementary Materials

The following are available online at https://www.mdpi.com/article/10.3390/brainsci11050606/s1, Table S1: Processing methodologies of recent fNIRS motor control studies, Table S2: Processing technique usage.
