# Supplementary material for: Data Processing in Functional Near-Infrared Spectroscopy (fNIRS) Motor Control Research
Source: Brain Sci. 2021 May 9;11(5):606. doi: 10.3390/brainsci11050606 (PMC8151801; doi:10.3390/brainsci11050606)
Supplement: Supplementary file 1 [file brainsci-11-00606-s001.zip › brainsci-1184790-supplementary.pdf]

Supplementary tables

Table S1: Processing Methodologies of Recent fNIRS Motor Control Studies.

Author/Date	Participant Demographics	Processing Techniques	Type of task
Almulla et al. 2020	n = 9 (2F, median age 36 yrs)	Applied bandpass filter (0.01-0.2 Hz), used GLM (canonical basis function)	Standing and sitting, ME + MI
Amemiya et al. 2010	n = 33 (18F, avg age 21.4 yrs)	Applied moving average to data smoothing factor of 5, block averaged data, used two-way repeated-measures ANOVA	Finger tapping ME + MI
Anwar et al. 2013	n = 6 (5F, avg age 25 yrs)	Bandpass filtered between 0.01-0.5 Hz, smoothed time series with window length of 25 points, unspecified analysis	Finger tapping ME
Anwar et al. 2016	n = 9 (5F, avg age 27 yrs)	Removed mean time series from time series, no filtering applied, used MVAR to model time series between fNIRS, fMRI, and EEG	Finger tapping ME
Asahara and Matsukawa 2018	n = 19 (7F, avg age 24 yrs)	Averaged data for every 5 seconds, used ANOVA	Cycling
Asahara et al. 2018	n = 13 (5F, avg age 25 yrs)	Sequentially averaged data for every 1 s, used ANOVA	Cycling
Aumen et al. 2020	n = 3 (0F, avg age 18 ± 2.65 yrs)	Applied GLM (canonical basis function, with autoregressive prewhitening algorithm, and SSC regression)	Dual task and dual task with wall toss
Bai et al. 2020	n = 15 (0F, avg age 26.93 yrs, SE = 0.71)	Applied spline interpolation, applied bandpass filter (0.01-0.2 Hz), block averaged, repeated measures ANOVA	Normal vision and mirrored with and without purdue pegboard task
Banville et al. 2017	n = 12 (5F, avg age 24.6 yrs)	Bandpass filtered 0.8-1.2 Hz, rejected channels with nonsignificant correlation across all epochs, bandpass filtered again 0.01-0.3 Hz, block averaged, used SVM classifier	Finger Tapping MI
Batula et al. 2017a	n = 13 (sex not reported, aged 18-35)	Applied 20th-order low-pass filter (FIR, 0.1 Hz), any channels with very high gain removed, CAR, used linear mixed models (assessed FDR)	U+L ME+MI
Batula et al. 2017b	n = 13 (sex not reported, aged 18-35)	100th order low-pass FIR filter (0.1 Hz), CAR, CBSI, used linear mixed models (assessed FDR)	U+L ME+MI
Beurskens et al. 2014	N = 25, n_1 = 15 (sex not reported, avg age 24.5 ± 3.3 yrs) and n_2 = 10 (sex not reported, avg	Applied moving standard deviation and spline interpolation, baseline-corrected, applied pre-coloring filter, applied wavelet-MDL de-trending algorithm, applied wavelet transform, used GLM (boxcar convolved with canonical)	Dual-task Walking

	age 71.0 ± 3.8 yrs)		
Brigadoi et al. 2012	n = 7 (0F, avg age 29.3 yrs)	Applied bandpass filter (0.01-0.3 Hz), applied algorithm (Scarpa et al 2011) to reduce global physiological noise, applied non-parametric Bayesian approach (Scarpa et al 2010) to reduce further, used ANOVA	Finger tapping ME
Bruno et al. 2018	n = 21 (10F, avg age 23.48 yrs)	Applied motion artifact correction with wavelet filter, applied bandpass filter (0.01-0.5 Hz), applied GLM (unspecified)	Driving
Buccino et al. 2016	n = 15 (0F, avg age 27.4 yrs)	Applied 4th order IIR Butterworth bandpass filter (0.01-0.2 Hz), mean + SD normalized, used LDA classifier	Arm task
Button et al. 2015	n = 32 (16F, aged 18-45 yrs)	Applied moving average (5.0s), used ANOVA-simultaneous component analysis	Swimming
Chacaroun et al. 2019	n = 21 (12F, avg age 29 yrs)	Applied Gaussian smoothing algorithm (2s width), used two-way ANOVA	Cycling
Chen et al. 2017	n = 90 (46F, avg age 78.1 yrs)	Applied low-pass filter (FIR, 0.14 Hz), used linear mixed effects models	Dual-task Walking
Choi et al. 2019	n = 18 (0F, avg age 25.3 yrs)	Applied low-pass filter (FIR, 0.2 Hz), used repeated-measures ANOVA	Balance task
Crivelli et al. 2018	n = 20 (10F, avg age 24.15 yrs)	Applied bandpass filter (0.01-0.3 Hz), used repeated-measures ANOVA	Upper Limb Tasks (22 actions)
De Lima Pardini et al. 2017	n = 8 (0F, avg age 23.63 yrs)	Downsampled (2 Hz), applied bandpass filter (FIR, 0.02-0.2 Hz), applied GLM (canonical boxcar function, discrete cosine transforms with a cutoff of 128s), applied precoloring	Walking
Derosiere et al. 2014	n = 15 (sex not reported, avg age 28 yrs)	Applied low-pass filter (0.7 Hz), detrended, block averaged, used two-way repeated-measures ANOVA	Hand grasping/clenching ME
Dresler et al. 2011	n = 6 (0F, avg age 29.6 yrs)	Applied low-pass 3rd order Butterworth filter (0.3 Hz), applied GLM (canonical boxcar function, cosine-filter with a cutoff of 35s)	Dreamed Hand Clenching
Ferrari et al. 2014	n = 22 (0F, avg age 26.5 yrs)	Detrended, applied low-pass filter (0.1 Hz), used mixed model ANOVA and repeated-measures ANOVA	Balance task
Fu et al. 2017	n = 6 (3F, avg age 26.8 yrs)	Applied low-pass filter (0.1 Hz), linearly detrended, block averaged, used ANOVA	Hand grasping/clenching MI

Funane et al. 2014	n = 14 (0F, avg age 40 yrs)	Applied low-pass filter (0.5 Hz), and high-pass filter (0.0125 Hz), applied Gaussian smoothing (FWHM: 2s), used MD-ICA	Finger tapping ME
Gagnon et al. 2012	n = 6 (sex not reported, age not reported)	Applied bandpass filter (0.01-1.25 Hz), applied Kalman filter to regress SS channels and recover HRF, applied Rauch-Tung-Striebel smoother, further applied low-pass filter (0.5 Hz), applied GLM (Gaussian functions, mean sep = 0.5s, SD = 0.5s)	Finger tapping ME
Groff et al. 2019	n = 10 (4F, avg age 22.1 yrs)	Applied moving average (5.0s), applied high-pass filter (0.01 Hz), unclear (correlation analysis with boxcar then block averaged)	Walking
Harrison et al. 2018	n = 17 (7F, aged 19-36 yrs)	Applied moving average (5.0s), applied high-pass filter (0.01 Hz), applied PCA, correlation analysis with boxcar, then averaging	Finger tapping ME
Heinze et al. 2019	n = 15 (5F, avg age 21.5 yrs)	Applied bandpass filter (0.01-0.2 Hz), used GLM (canonical boxcar function)	Learning Piano Chords
Hernandez et al. 2020	n = 20 (15F), n1 = 12 (8F, avg age 66.7 ± 5.4 yrs), n2 = 8 (7F, avg age 22.9 ± 2.8 yrs)	Applied low-pass filter (0.14 Hz), applied wavelet filter, used repeated measures ANOVA	Walking, dual task walking
Herold et al. 2017	n = 10 (sex not reported, aged 21-47 yrs)	Applied a moving average (5.0 s), applied low-pass filter (0.5 Hz), block averaged and t-test (FDR corrected)	Balance task
Herold et al. 2019	n = 13 (sex not reported, age not reported)	Applied wavelet (IQR = 1.219) and bandpass filters (0.01-0.5 Hz), used Wilcoxon tests (FDR corrected)	Walking
Holper and Wolf 2010	n = 15 (9F, avg age 29 yrs)	Applied low-pass filter (0.1 Hz), smoothed with 1st order Savitzky-Golay filter (501 points), used Wilcoxon test, then one-way ANOVA	Finger tapping ME + MI
Holper and Wolf 2011	n = 12 (6F, avg age 29 yrs)	Applied low-pass 7th order Chebyshew filter (20dB attenuation at 5 Hz), downsampled to 10 Hz, used paired t-test	Finger tapping ME + MI
Holper et al. 2010	n = 23 (sex not reported, age not reported)	Applied low-pass 7th order Chebyshew filter (20dB attenuation at 5 Hz), downsampled to 10 Hz, used one-way ANOVA	Observation/VR/VMT + MI
Holper et al. 2012	n = 17 (10F, avg age 25.6 yrs)	Applied low-pass 7th order Chebyshew filter (20dB attenuation at 5 Hz), downsampled to 10 Hz, applied spline interpolation for motion artifacts, used repeated-measures ANOVA	Observation/VR/VMT + MI
Holper et al. 2014	n = 17 (7F, avg age 30.7 yrs)	Applied low-pass 7th order Chebyshew filter (20dB attenuation at 5 Hz), downsampled to 10 Hz, applied spline interpolation for motion artifacts, used multivariate ANOVA	Finger tapping ME

Holtzer et al. 2011	N = 22, n_1 = 11 older adults (7F, aged 69-88 yrs) and n_2 = 11 younger adults (7F, aged 19-29 yrs)	Applied low-pass filter (FIR, 0.14 Hz), applied combined ICA/PCA, used linear mixed effects models	Dual-task Walking
Holtzer et al. 2017	n = 314 (56F, avg age 76.8 ± 6.7 yrs)	Applied low-pass filter (FIR, 0.14 Hz), used linear mixed effects model	Dual-task Walking
Holtzer et al. 2019	n = 75, n_1 = 19 with fear of falling (13F, avg age 79.84 ± 6.01 yrs) and n_2 = 56 without fear of falling (25F, avg age 76.73 ± 6.39 yrs)	Applied low-pass filter (FIR, 0.14 Hz), linear mixed effects models	Dual-task Walking
Holtzer. et al. 2020	n = 394 (217F, avg age 76.29 ± 6.65 yrs)	Applied wavelet filter (Daubechies 5), applied spline interpolation, applied low-pass filter (0.08 Hz), baseline corrected, used linear mixed-effects models	Dual-task walking
Hong and Naseer 2016	n = 2 (0F, age not reported)	Applied low-pass filter (0.3 Hz), applied high-pass filter (0.01 Hz), used ARMAX	Hand grasping/clenching ME
Hu et al. 2013	n = 5 (0F, aged 24-31 yrs)	Applied bandpass filter (0.01-0.08 Hz, Butterworth), applied GLM (gamma function)	Finger tapping ME
Huppert et al. 2013	n = 10 (5F, aged 21-47 yrs)	Applied GLM (canonical gamma functions, discrete cosine transform (0-1/120 Hz)), prewhitened data (2nd order autoregressive)	Walking
Ishii et al. 2018	n = 22 (11F, avg age 25 yrs)	Downsampled to 1Hz, block averaged and used one-way ANOVA	Arm task
Jang et al. 2014	n = 12 (5F, avg age 29 yrs)	Applied Gaussian smoothing (FWHM = 2s), applied wavelet-MDL based detrending, applied GLM (canonical HRF)	Hand grasping/clenching ME
Jin et al. 2018	n = 22 (5F, avg age 22 yrs)	Applied wavelet decomposition, then applied power spectrum density analysis	Walking
Karim et al. 2012	n = 9 (4F, aged 18-42 yrs)	Applied GLM (canonical boxcar function, discrete cosine transform 0-1/120 Hz), prewhitening (2nd order autoregressive)	Balance task
Khan et al. 2018	n = 9(0F, avg age 30 yrs)	Applied low-pass filter (0.5 Hz), applied high-pass filter (0.01 Hz), compared different filters (Butterworth, Kalman, HRF, FIR, Wiener, Gaussian), used multiple classifiers (SVM, NB, LDA, QDA, KNN)	Walking
Kim et al. 2017	n = 1 (1F, aged 30 yrs)	Applied Gaussian smoothing (FWHM = 2s), used wavelet detrending for motion artifacts, applied GLM (canonical)	Walking

Kobashi et al. 2012	n = 12 (13F, avg age 25.6 yrs)	Applied spline interpolation to reduce motion artifacts, low-pass filter (7th order Chebyshew with 20dB attenuation @ 5 Hz), downsampled to 10 Hz, used two-way repeated-measures ANOVA	Observation/VR/VMT + MI
Koehler et al. 2012	n = 39 (25F, avg age 21.8 yrs)	Applied moving average (5s), block averaged and used t-tests	Observation/VR/VMT ME
Koenraadt et al. 2012	n = 13 (11F, avg age 24 yrs)	Applied low-pass filter (Butterworth, 1 Hz), block averaged, converted to CoGs, used two-way repeated-measures ANOVA	Hand + Foot Tapping
Koenraadt et al. 2013	n = 11 (9F, avg age 30 yrs)	Applied low-pass filter (2nd order Butterworth, 1 Hz), used t-tests	Finger tapping ME
Koenraadt et al. 2014	n = 11 (8F, avg age 23 yrs)	Applied low-pass filter (2nd order Butterworth, 1.25 Hz), applied high-pass filter (2nd order Butterworth, 0.01 Hz), regressed SS channels, applied low-pass filter again (2nd order Butterworth, 1 Hz), used one-way repeated-measures ANOVA	Walking
Koren et al. 2019	n = 20 (sex not reported, aged 18-30 yrs)	Applied spline interpolation to correct motion artifacts (p = 0.99), applied wavelet correction as needed for motion artifacts (IQR = 0.1), applied bandpass filter (0.01-0.2 Hz), used linear mixed effects model	Obstacle Negotiation
Kotegawa. et al. 2020	n = 15 (7F, avg age 21.7 ± 4.4 yrs)	Bandpass filtered (0.01-0.1 Hz), standardized to a z score, block averaged, used two-way ANOVA	Walking (ME + MI)
Kurz et al. 2012	n = 13 (sex not reported, avg age 23.7 yrs)	Applied high-pass filter (0.01 Hz), applied 5s moving average, applied PCA on all channels, correlation analysis (boxcar) and block averaged	Walking
Lachert et al. 2017	n = 10 (5F, avg age 28.1 yrs)	Detrended via linear approximation, smoothed with a moving average (10 points), applied low-pass filter (0.4 Hz), calculated z-scores	Finger tapping ME
Lee et al. 2020	n = 10 (5F, avg age 22.7 ± 3.2 yrs)	Applied low-pass filter (2 Hz), rejected channels with SNR < 30 dB, used movement artifact reduction algorithm (MARA) to reduce motion noise, applied bandpass filter (0.02-0.2 Hz), baseline-corrected, applied a global regression to reduce noise, used an ANOVA	Walking
Li C et al. 2020a	n = 30 (14F, avg age 21 ± 1 yrs)	Applied low-pass filter (2nd order Chebyshev, 0.145 Hz), applied a mathematical morphology filter (MMF), used entropy weight method (EWM) to calculate ROI signals and remove motion artifacts	Walking (ME + MI)
Li C et al. 2020b	n = 30 (14F)	Applied low-pass filter (Chebyshev)	Walking
Li C et al. 2020c	n = 30 (14F, avg age 21 ± 1 yrs)	Applied low-pass filter (2nd order Chebyshev, 0.145 Hz), then applied a MMF	Walking

Li X et al. 2020	n = 21 (12F, 19RH, avg age 33.5 ± 15.5 yrs)	Applied spline-SG (spline interpolation and Savitsky-Golay filter combination) to remove motion artifacts, applied bandpass filter (6th order Butterworth, 0.01-0.5 Hz), applied GLM (OLS, Gaussian basis functions, used SSC regressor	ME and observation
Lin et al. 2012	n = 25, n_1 = 12 younger (4F, avg age 23.4 yrs) and n_2 = 13 elderly (6F, avg age 67.6 yrs)	Applied 3rd order IIR Butterworth bandpass filter (0.002-0.5 Hz), applied PCA, block averaged, used two-way repeated-measures ANOVA	Cycling
Lin et al. 2016	n = 19 (0F, avg age 23.7 yrs)	Applied 3rd order IIR Butterworth bandpass filter (0.0016-0.8 Hz), block averaged and used repeated-measures ANOVA	Knee Extension
Lu et al. 2013	n = 10 (5F, avg age 22.7 yrs)	Applied low-pass filter (0.5 Hz), applied GLM (unspecified)	Hand grasping/clenching ME
Lu et al. 2015	n = 17 (8F, avg age 23.1 yrs)	Applied bandpass filter (0.01-0.2 Hz), applied PCA and spike rejection, block averaged and used t-tests	Dual-task Walking
Lu et al. 2017	n = 29 (15F, avg age 24.2 yrs)	Applied bandpass filter (0.1-0.8 Hz), used GLM with canonical boxcar function	Finger tapping ME
Lucas et al. 2019	n = 55 (27F, avg age 74.76 yrs)	Applied low-pass filter (0.14 Hz) , used linear mixed effects model	Dual-task Walking
Maidan et al. 2018	n = 20 (sex not reported, aged 20-50 yrs)	Applied bandpass (0.01-0.14 Hz), applied wavelet filter, applied CBSI, used two linear mixed effects models	Obstacle Negotiation
Mandrick et al. 2013	n = 15<br><br>(0F, avg age 28.3 ± 6 yrs)	Applied low-pass filter (0.1 Hz), utilized time series analysis technique (amplitude-based approach), used two-way ANOVA	Hand grasping/clenching ME
Mehnert et al. 2013	n = 20 (5F, avg age 27.7 yrs)	Linearly interpolated data to correct for motion artifacts, applied 3rd order Butterworth bandpass filter (0.016-0.2 Hz), applied GLM (canonical basis)	Hand/finger movements ME
Mehta and Rhee 2017	n = 9 (6F, aged 65 yrs or older)<br><br>n = 10 (6F, aged 20–35 yrs)	Applied low-pass filter (3 Hz), used wavelet-based method (1.5 IQR), applied low-pass filter again (0.5 Hz), applied high-pass filter (0.016 Hz), used mixed factor ANOVA	Arm task
Metzger et al. 2017	n = 12 (8F, avg age 27.6 yrs)	Applied moving average within a time window of five seconds, baseline corrected (10s), linear fit (consisting of 10-s baseline, 45-s activation task, and 15-s rest), applied CBSI, used t-tests, then a 3x2 repeated-measures ANOVA	Dual-task Walking

Mirelman et al. 2014	n = 23 (13F, avg age 30.9 ± 3.7 yrs)	Applied low-pass filter with a finite impulse response filter (0.14 Hz), applied Continuous Wavelet transform, used repeated-measures ANOVA	Dual-task Walking
Mirelman et al. 2017	N = 43, n_1 = 23 young adults (13F, avg age 30.9 ± 3.7 yrs) and n_2 = 20 older adults (10F, avg age 69.7 ± 5.8 yrs)	Applied bandpass filter (0.01–0.14 Hz), applied wavelet filter, applied CBSI, used linear mixed model	Dual-task Walking
Moro et al. 2016	n = 21 (0F, avg age 26.1 yrs)	Applied wavelet filter (iqr = 1) for motion artifact correction, applied a deconvolution GLM (temporal basis = gaussian (2s apart, SD = 3s)) which also used SS channels to regress physiological signal, then IRLS method to solve GLM	Observation/VR/VMT ME
Muthalib et al. 2015	n = 9 (0F, avg age 39.2 ± 13 yrs)	Applied GLM (canonical), detrending filter (Wavelet-MDL), and Time series (O2Hb or HHb time course) modeled as a linear combination of L regressors	Wrist Extension ME
Muthalib et al. 2016	n = 8 (sex not reported, avg age 30.4 ± 10.6 yrs)	Applied low-pass filter (0.1 Hz), baseline corrected (last 5 seconds of rest), block averaged and used repeated-measures ANOVA	Finger tapping ME
Muthalib et al. 2018	n = 8 (0F, avg age 25.9 ± 4.3 yrs)	Applied low-pass filter (0.1 Hz), block averaged and used repeated-measures ANOVA	Wrist Extension ME
Nagasawa et al. 2020	n = 9 (1F, 21-26 yrs)	Detrended (discrete cosine transform), baseline corrected, applied moving average (window = 3s)	Hand task ME
Naseer and Hong 2013	n = 10 (0F, avg age 28.5 ± 4.8 yrs)	Baseline corrected, applied low-pass filter using 4th order Butterworth filter (0.1 Hz), used LDA classifier	Wrist Extension MI
Nazeer et al. 2020a	n = 7 (0F, avg age 27 ± 5 yrs)	Bandpass filter (0.01–0.3 Hz), hemodynamic response filter, discrete cosine transforms	Finger-tapping ME
Nazeer et al. 2020b	N = 29 (15 F avg age 28.5 ± 3.7 yrs)	Bandpass filtered using fourth-order Butterworth filter (0.03–0.15 Hz), smoothing (Savitzky–Golay filter)	Mental arithmetic (MA) and motor imagery (MI)
Novi et al. 2020	n = 10 (0F, avg age 22 ± 5 yrs)	Low signal-to-noise ratio (SNR) removed based on SNR < 8, spline interpolation, wavelet decomposition, bandpass filter (0.005-0.5 Hz)	Finger-tapping ME
Peters et al. 2020	n = 14 (7F, avg age 34 ± 8 yrs)	Applied wavelet transformation (1.5), low-pass filter (0.15 Hz), and GLM (ordinary least squares) with SSC	Walking in a robotic exoskeleton

Pfurtscheller et al. 2011	n = 26 (14F, avg age 23 ± 2.8 yrs)	Applied 5th-order low-pass Butterworth filter (0.9 Hz), used paired-sample t-tests	Hand/finger movements ME
Pittaccio et al. 2013	n = 2 (0F, avg age 32 ± 7.1 yrs)	Applied low pass filter (0.030 Hz), segmented continuous tracks into epochs, applied GLM (unspecified)	Ankle Dorsiflexion
Polskaia et al., 2020	n = 17 (avg age 23.25±2.21 yrs)	Fourth-order Butterworth bandpass filter (0.1-0.5), principle component analysis (PCA)	Finger-tapping ME
Propper et al. 2017	n = 39 (0F, ages not reported)	Applied finite impulse response filter, applied cut-off between 400 mV - 4000 mV, used a 2x3 mixed ANOVA	Hand grasping/clenching ME
Rahimpour et al. 2020	n = 10 (0F, 22.5)	Autoregressive iterative reweighted least square (AR-IRLS) algorithm, pre-whitening, robust regression	Finger-tapping ME
Rosner and Barlow 2016	n = 22 (17F, avg age 23.16 ± 1.76 yrs)	Applied low-pass filter (0.3 Hz), applied automated detection algorithm based on standard deviation, block averaged from -10s to +30s, then used ANOVA	Hand grasping/clenching ME
Rosso et al. 2017	N = 16, n_1 = 10 (7F, aged 66–81 yrs), n_2 = 6 (2F, aged 22–30 yrs)	Applied GLM (canonical) with autoregressive prewhitening filter, solved using IRLS	Balance task
Sagari et al. 2015	n = 20 (11F, avg age 27.5 ± 5.5 yrs)	Applied moving average (window: 5 s), block averaged, used Spearman's rank correlation coefficients	Character Entry
Sagari et al. 2020	n = 13 (11F, avg age, 21.4 ± 1.0 yrs)	Moving average	Upper and lower limb tasks
Schurholz et al. 2012	n = 8 (6F, avg age 24.8±2.4 yrs)	Detrended (of global trends and of noise components) using wavelet-MDL, utilized precoloring in GLM (unspecified)	Wrist Flexion/Extension MI
Seidel et al. 2017	n = 43 (20F, avg age 25 yrs)	Signal quality checked by CV (15% cutoff) applied baseline correction (10s of rest), applied bandpass filter (0.01 Hz to 0.2 Hz), applied time series analysis, applied GLM (unspecified)	Balance task
Seidel et al. 2019	n = 42 (16F, avg age 26.81 yrs)	Signal quality checked by CV (15% cutoff), applied wavelet filtering (1.219 IQR), applied bandpass filter (0.01-0.5 Hz), applied short separation regression in GLM (Gaussian functions), solved using OLS	Cycling

Shibuya 2011	n = 12 (0F, avg age 24.08 ± 0.18 yrs)	Baseline corrected (30s), applied high-pass filter (0.02 Hz), used repeated-measures ANOVA	Hand grasping/clenching ME
Shibuya et al. 2014	n = 10 (0F, avg age 21.1 ± 0.84 yrs)	Applied low-pass filter (0.7 Hz), baseline corrected (60s), block averaged and used a repeated-measures ANOVA	Hand/finger movements ME
Shibuya et al. 2016	n = 10 (0F, avg age 22.2 ± 0.2 yrs)	Applied CAR, projected onto CSP, used repeated-measures ANOVA	Hand grasping/clenching ME
Shin et al. 2014	n = 8 (1F, avg age 26.8 ± 1.6 yrs)	Normalized, applied zero-phase distortion filter (third-order bandpass Chebyshev I with a ripple factor of 0.5 dB; 0.03-0.07 Hz), applied CBSI, applied third-order Savitzky–Golay smoothing filter, used NB classification	Arm Lifting + Knee Extension
Stuart et al. 2019	n = 35, n_1 = 17 (9F, avg age 20.3 ± 1.2 yrs), n_2 = 18 (9F, avg age 72.6 ± 8.0 yrs)	Applied low-pass filter (0.15 Hz), applied wavelet-minimum description length algorithm, applied baseline correction, block averaged and used linear mixed effects models	Dual-task Walking
Tempest et al. 2019	n = 13 (7F, avg age 29.8 ± 3.9 yrs)	Pruned channels with poor signal quality, applied wavelet-based motion correction, applied bandpass filter (0.01-0.5 Hz), applied GLM (unspecified)	Hand grasping/clenching ME
Urquhart et al. 2020	n = 23 (4F, 25.13 ± 3.72 yrs)	Detrended with least-squares fit, low-pass filtered with third order Butterworth filter, high-pass filtered with fifth order Butterworth, channels were removed if signal was > 2 SD mean amplitude, principal component analysis (PCA)	Hand grasping
Vasta et al. 2017	n = 11 (5F, aged 27–64 yrs)	Applied moving average with a window width of 5 s, applied bandpass filter (0.005-0.5 Hz), used generalized linear model analyses of variance	Hand/finger movements ME
Waldert et al. 2012	n = 17 (7F, avg age 26.8 ± 5.6 yrs)	Applied low-pass filter using a 3rd order Butterworth filter, applied a causal filtering (zero phaseshift) with 0.12 Hz cutoff, used regularized LDA classifier	Hand/finger movements ME
Willis et al. 2019	n = 7 (2F, avg age 26.6 ± 2.9 yrs)	Applied fourth-order low-pass zero-phase Butterworth filter (0.2 Hz), used linear mixed effects model	Sprinting
Wolf et al. 2011	n = 12 (1F, avg age 28.8 ± 12.7 yrs)	Applied DPF-method, applied low-pass filter (0.1 Hz), used Pearson's correlation coefficient and Spearman's Rho	Hand grasping/clenching ME

Woorons et al. 2019	n = 10 (3F, avg age 19.2 ± 2.3 yrs)	Applied low-pass filter (0.1 Hz), used oxygenation index (difference between HbO and HbR), used two-way repeated-measures ANOVA	Sprinting
Wriessnegger et al. 2017	n = 13 (5F, avg age 25 ± 3 yrs)	Applied high-pass filter (0.01 Hz), applied low-pass filter (0.8 Hz), utilized TF models, block averaged, used t-tests and ANOVA	Hand grasping/clenching ME + MI
Wriessnegger et al. 2018	n = 20 (10F, avg age 24.8 ± 2.5 yrs)	Applied high-pass filter (0.01 Hz), applied low-pass filter (0.8 Hz), utilized TF models, applied spatial smoothing, used 5x6x5 repeated-measures ANOVA	Hand grasping/clenching MI
Xu et al. 2020	n = 36 (16F, avg age 20.6 ± 1.5 yrs)	Spikes were manually deleted and bandpass filter (0.01-0.1 Hz)	Table-setting tasks
Yang et al. 2020	n = 12 (7F, avg age 32.5 ± 9.4 yrs)	Signal to noise ratio (SNR) values < 3 were excluded, spline-SG, low-pass filter (0.5 Hz), GLM (ordinary least square method), baseline drift corrected with third order polynomial fit, and short separation channel (SSC)	Shoulder abduction and finger extension
Yeo et al. 2013	n = 9 (2F, avg age 27.4 ± 3.2 yrs)	Applied Gaussian smoothing (FWHM = 2s), applied wavelet-MDL based detrending algorithm, used t-tests	Arm task
Yin et al. 2015	n = 6 (3F, avg age 26.8 ± 3.3 yrs)	Applied linear-detrending filter, applied low-pass 5th-order Chebyshev II filter (0.1 Hz), downsampled to 1 Hz, used ELM classifier	Hand grasping/clenching ME + MI
Yokoyama et al. 2019	n = 20 (20F, avg age 21.6 yrs)	Applied moving average (smoothing factor of 25 points), applied baseline normalization (-5s to 0s), used two-way ANOVA	Hand grasping/clenching ME
Yozu et al. 2016	n = 10 (5F, avg age 32.0 ± 7.7 yrs)	Applied baseline drift correction, applied first-degree baseline fit, averaged across channels over SMA, block averaged and used paired t-tests	Quadrapedalism + Walking
Yu et al. 2014	n = 8 (sex not reported, age not reported)	Applied low-pass filter, applied GLM (unspecified) and Sun's tube formula	Hand grasping/clenching MI
Zafar & Hong, 2020	n = 11 (unspecified)	Applied fourth-order Butterworth low- and high-pass filters (0.15 and 0.01 Hz)	Fingering tapping ME
Zimmermann et al. 2013	n = 7 (1F, avg age 26.0 ± 2.2 yrs)	Downsampled to 5 Hz, applied 5-point median filter, used inverse z-score of the raw intensity signals to identify MA, used a HMM classifier	Hand/finger movements ME

Table S2: Processing Technique Usage

Processing Techniques	Used by
Band-pass, Low-pass, and High-pass filtering	Almulla et al. 2020; Anwar et al. 2013; Bai et al. 2020; Banville et al. 2017; Batula et al. 2017a, 2017b; Brigadoi et al. 2012; Bruno et al. 2018; Buccino et al. 2016; Chen et al. 2017; Choi et al. 2019; Crivelli et al. 2018; de Lima-Pardini et al. 2017; Derosiere et al. 2014; Dresler et al. 2011; Ferrari et al. 2014; Fu et al. 2017; Funane et al. 2014; Gagnon et al. 2012; Groff et al. 2019; Harrison et al. 2018; Heinze et al. 2019; Hernandez et al. 2020; Herold et al. 2017, 2019; Holper and Wolf 2010, 2011; Holper et al. 2010, 2012, 2014; Holtzer et al. 2011, 2017, 2019, 2020; Hong and Naseer 2016; Hu et al. 2013; Khan et al. 2018; Kobashi et al. 2012; Koenraadt et al. 2012, 2013, and 2014; Koren et al. 2019; Kotegawa et al. 2020; Kurz et al. 2012; Lachert et al. 2017; Lee et al. 2020; Li C et al. 2020a, 2020b, 2020c; Li X et al. 2020; Lin et al. 2012, 2016; Lu et al. 2013, 2015, 2017; Lucas et al. 2019; Maidan et al. 2018; Mandrick et al. 2013; Mehnert et al. 2013; Mehta and Rhee 2017; Mirelman et al. 2014, 2017; Muthalib et al. 2016, 2018; Naseer and Hong 2013; Nazeer et al. 2020a, 2020b; Novi et al. 2020; Peters et al. 2020; Pfurtscheller et al. 2011; Pittaccio et al. 2013; Polskaia et al. 2020; Propper et al. 2017; Rosner and Barlow 2016; Seidel et al. 2017, 2019; Shibuya 2011; Shibuya et al. 2014; Shin et al. 2014; Stuart et al. 2019; Tempest et al. 2019; Urquhart et al. 2020; Vasta et al. 2017; Waldert et al. 2012; Willis et al. 2019; Wolf et al. 2011; Woorons et al. 2019; Wriessnegger et al. 2017, 2018; Xu et al. 2020; Yang et al. 2020; Yin et al. 2015; Yu et al. 2014; Zafar and Hong 2020
Smoothing (moving average, Savitzky-Golay filter, Gaussian weighted filter)	Amemiya et al. 2010; Anwar et al. 2013; Chacaroun et al. 2019; Funane et al. 2014; Gagnon et al. 2012; Holper and Wolf 2010; Jang et al. 2014; Koehler et al. 2012; Kim et al. 2017; Lachert et al. 2017; Nagasawa et al. 2020; Nazeer et al. 2020b; Sagari et al. 2015, 2020; Shin et al. 2014; Wriessnegger et al. 2018; Yang et al. 2020; Yeo et al. 2013; Yokoyama et al. 2019
Downsampling	De Lima-Pardini et al. 2017; Holper and Wolf 2011; Holper et al. 2010, 2012, 2014; Ishii et al. 2018; Kobashi et al. 2012; Matsukawa et al. 2020; Yin et al. 2015; Zimmermann et al. 2013

Wavelet filtering	Beurskens et al. 2014; Bruno et al. 2018; Hernandez et al. 2020; Herold et al. 2019; Holtzer et al. 2020; Jang et al. 2014; Jin et al. 2018; Kim et al. 2017; Koren et al. 2019; Maidan et al. 2018; Mehta and Rhee 2017; Mirelman et al. 2014, 2017; Moro et al. 2016; Muthalib et al. 2015; Novi et al. 2020; Peters et al. 2020; Schurholz et al. 2012; Seidel et al. 2019; Stuart et al. 2019; Tempest et al. 2019; Yeo et al. 2013
PCA Filter	Harrison et al. 2018; Holtzer et al. 2011; Kurz et al. 2012; Lin et al. 2012; Lu et al. 2015; Polskaia et al. 2020; Urquhart et al. 2020
Independent Component Analysis (ICA) Filter	Holtzer et al. 2011
General Linear Model (GLM)	Almulla et al. 2020; Aumen et al. 2020; Bruno et al. 2018; de Lima-Pardini et al. 2017; Dresler et al. 2011; Gagnon et al. 2012; Hu et al. 2013; Huppert et al. 2013; Jang et al. 2014; Karim et al. 2012; Kim et al. 2017; Li X et al. 2020; Lu et al. 2013; Mehnert et al. 2013; Moro et al. 2016; Muthalib et al. 2015; Peters et al. 2020; Pittaccio et al. 2013; Rahimpour et al. 2020; Rosso et al. 2017; Seidel et al. 2017, 2019; Tempest et al. 2019; Yang et al. 2020; Yu et al. 2014
Kalman filtering	Gagnon et al. 2012; Khan et al. 2018
Standard Deviation Cutoff	Beurskens et al. 2014; Rosner and Barlow 2016
Inverse Z-score	Zimmermann et al. 2013
Linear Interpolation	Fu et al. 2017; Lachert et al. 2017; Mehnert et al. 2013; Metzger et al. 2017; Yin et al. 2015
Spline Interpolation	Bai et al. 2020; Beurskens et al. 2014; Holper et al. 2012, 2014; Holtzer et al. 2020; Kobashi et al. 2012; Koren et al. 2019; Novi et al. 2020
Common Average Referencing (CAR)	Batula et al. 2017a, 2017b; Shibuya et al. 2016
Correlation-Based Signal Improvement (CBSI)	Batula et al. 2017b; Maidan et al. 2018; Metzger et al. 2017; Mirelman et al. 2017; Shin et al. 2014
Prewhitening	Aumen et al. 2020; Huppert et al. 2013; Karim et al. 2012; Rahimpour et al. 2020
Precoloring	Beurskens et al. 2014; De Lima-Pardini et al. 2017; Schurholz et al. 2012

Bayesian modelling	Brigadoi et al. 2012
Short-Separation Channel Regression	Aumen et al. 2020; Gagnon et al. 2012; Koenraadt et al. 2014; Li X et al. 2020; Moro et al. 2016; Peters et al. 2020; Seidel et al. 2019; Yang et al. 2020
Block Averaging	Bai et al. 2020; Kotegawa et al. 2020
Linear Mixed Models	Holtzer et al. 2020
Movement Artifact Reduction Algorithm (MARA)	Lee et al. 2020
Mathematical Morphology Filter (MMF)	Li C et al. 2020a; Li C et al. 2020c
Entropy Weight Method (EWM)	Li C et al. 2020a
Spline Interpolation and Savitsky-Golay filter combination (Spline-SG)	Li X et al. 2020; Yang et al. 2020
